# Photonic Crystal Stimuli-Responsive Chromatic Sensors: A Short Review

**DOI:** 10.3390/mi11030290

**Published:** 2020-03-10

**Authors:** Andrea Chiappini, Lam Thi Ngoc Tran, Pablo Marco Trejo-García, Lidia Zur, Anna Lukowiak, Maurizio Ferrari, Giancarlo C. Righini

**Affiliations:** 1 Institute of Photonics and Nanotechnologies (IFN-CNR) CSMFO Laboratory and Fondazione Bruno Kessler (FBK) Photonics Unit, 38123 Povo (Trento), Italy; andrea.chiappini@ifn.cnr.it (A.C.); pablo.trejogarcia@alumno.buap.mx (P.M.T.-G.); lidia.zur@ifn.cnr.it (L.Z.); maurizio.ferrari@ifn.cnr.it (M.F.); 2 Department of Materials Technology, Faculty of Applied Sciences, Ho Chi Minh City University of Technology and Education, Ho Chi Min City 70000, Vietnam; lamttn@hcmute.edu.vn; 3 Faculty of Physico-Mathematical Sciences, Benemérita Universidad Autónoma de Puebla (BUAP), Puebla 72570, Mexico; 4 Institute of Low Temperature and Structure Research, PAS, 50-422 Wroclaw, Poland; a.lukowiak@intibs.pl; 5 Nello Carrara Institute of Applied Physics (IFAC CNR), 50019 Sesto Fiorentino (Firenze), Italy

**Keywords:** photonic crystal, optical sensing, nanostructures, mechanochromic sensors

## Abstract

Photonic crystals (PhC) are spatially ordered structures with lattice parameters comparable to the wavelength of propagating light. Their geometrical and refractive index features lead to an energy band structure for photons, which may allow or forbid the propagation of electromagnetic waves in a limited frequency range. These unique properties have attracted much attention for both theoretical and applied research. Devices such as high-reflection omnidirectional mirrors, low-loss waveguides, and high- and low-reflection coatings have been demonstrated, and several application areas have been explored, from optical communications and color displays to energy harvest and sensors. In this latter area, photonic crystal fibers (PCF) have proven to be very suitable for the development of highly performing sensors, but one-dimensional (1D), two-dimensional (2D) and three-dimensional (3D) PhCs have been successfully employed, too. The working principle of most PhC sensors is based on the fact that any physical phenomenon which affects the periodicity and the refractive index of the PhC structure induces changes in the intensity and spectral characteristics of the reflected, transmitted or diffracted light; thus, optical measurements allow one to sense, for instance, temperature, pressure, strain, chemical parameters, like pH and ionic strength, and the presence of chemical or biological elements. In the present article, after a brief general introduction, we present a review of the state of the art of PhC sensors, with particular reference to our own results in the field of mechanochromic sensors. We believe that PhC sensors based on changes of structural color and mechanochromic effect are able to provide a promising, technologically simple, low-cost platform for further developing devices and functionalities.

## 1. Introduction

**Figure 1:**
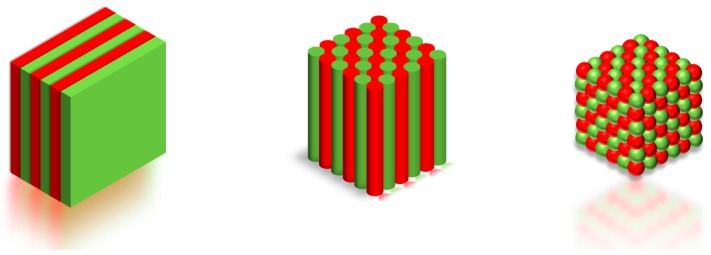
Sketch of the representation of the three kinds of photonic crystals. The different color represents materials with different dielectric constants. Adapted with permission from [1].

Photonic bandgap (PBG) crystals, often referred to simply as photonic crystals, are materials characterized by the periodic modulation of the dielectric constant along one, two, or three directions of space. Correspondingly, one speaks about one-, two-, and three-dimensional (1D, 2D and 3D, respectively) photonic crystals (see Figure 1). 

One can think about the interference of light scattered from lattice planes which may lead to the result that some frequencies are not allowed to propagate, giving rise to forbidden and allowed bands. Strictly speaking, however, the two terms PBG and PhC should not be considered as equivalent, since the latter (PhC) actually applies to any periodic dielectric or metallic structure, irrespective of the presence of a full photonic band gap. 

What is interesting is the similarity of the periodic modulation of the refractive index in a PhC with the atomic lattice in a solid; hence, the behavior of photons in a PhC is similar to electron and hole behavior in an atomic lattice. From a theoretical viewpoint, the determination of the eigenfunctions (or resonant frequencies) in a PhC is very similar to the calculation of the particle wave functions in the solid-state; in this way one can compute and design the photonic band structure.

Many structures and applications have been explored since the milestone papers on PhCs by Yablonovitch [2] and John [3], that followed the pioneering works on spontaneous emission control in 1D structures by Bykov [4,5] and on the concept of 3D photonic crystals by Ohtaka [6]. Maybe the simplest example of 1D PhC is represented by the Bragg grating, namely a layered structure made of alternating high-index and low-index films; the 1D structures are widely used as antireflecting coatings or highly reflective mirrors in certain laser cavities. The periodicity of the permittivity along two directions allows a larger variety of configurations: A periodic arrangement of circular holes in a silicon substrate or of dielectric rods in air provides two good examples of 2D PhCs. Thanks to the noticeable advances in Si-based systems, the integration of 2D PhCs with electronic circuits is now within reach. Much interest, however, is focused on the design of new geometric configurations of 3D PhCs, which may pave the way to the development of novel devices. It is clear that the ability to completely control the emission and the propagation of light simultaneously in all three dimensions would represent a disrupting achievement in the field of photonics. Fabrication of 3D PhCs, however, is a tough and challenging issue, even if laser direct writing, laser lithography and self-assembly methods have already produced valuable results. The reader interested in a deeper knowledge of the theory and applications of PhCs is referred to some of the many existing books [7,8,9,10,11,12,13,14,15]. 

A relevant field of application of PhCs is sensing, and design and fabrication methods and techniques have been developed, leading to feasibility demonstrations. Still, at least to our knowledge, no commercial PhC-based sensors exist, even if, in addition to journals’ publications, a number of MSc and PhD thesis works on this subject contribute to testify the research efforts in this direction of academic groups, too; see, for instance [16,17,18,19,20,21,22,23].

Many sensors based on photonic crystal fibers have also been demonstrated (see [24,25] for recent reviews), but they will not be considered here. We aim to provide a brief overview, even if far from being comprehensive, of the recent advances in the field of PhC sensors, with particular attention to the category of mechanochromic PhC sensors, that appear promising, especially due to their intrinsic characteristic of allowing a simple and low-cost optical readout. In fact, recent literature reports a large number of impressive results in this area, so that we considered worthy to write a short review, addressing the research efforts in the last decade on sensors responsive to three types of external stimuli: chemical, biological and physical. These sensors may have an impact on our daily lives, ranging from environmental aspects to tumor screening markers or drug delivery, and to structural health monitoring. 

For the sake of clarity, it may be noted that traditional sensors based on spectroscopic techniques (such as Raman, Brillouin, mass spectroscopy, etc.) are capable of a better performance in terms of sensitivity, whereas the new sensors based on chromatic response exhibit advantages in their fast detection, reversibility, low cost materials, and fabrication procedures; these aspects also make the fabrication of disposable chromatic sensors viable from the economic point of view.

It is also worth mentioning that in the present review we have focused attention on low refractive index contrast PhCs, based on glassy materials, that can be easily obtained exploiting sub-micrometric periodic structures that strongly affect the light matter interaction.

Section 2 provides a brief description of how the color appearance of a PhC structure, including several natural living organisms, is correlated to the geometrical and refractive index characteristics of the structure itself, and how this correlation may be exploited for sensing applications. Section 3 is devoted to the detection of chemical species, e.g., analytes in liquid or gaseous phases, by PhCs with different dimensionalities, whereas Section 4 describes different PhCs sensitive to biological species, which proved to be able to reveal tumor markers and biomolecules. Section 5 reports some specific results concerning responsive materials and mechanochromic structures, capable to detect mechanic stimuli. The Conclusions section will briefly outline the current trends and some prospects of photonic crystal sensors. 

## 2. Natural Photonic Crystals and Structural Colors

**Figure 2:**
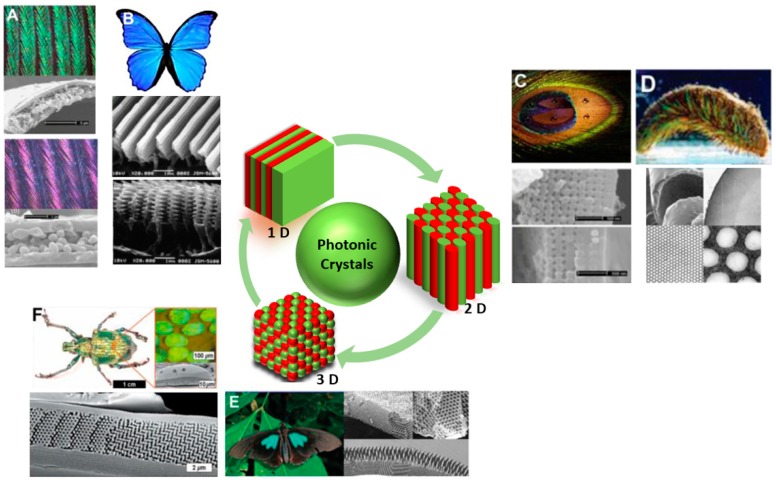
Several examples of natural photonic crystals with various structural colors. (**A**) One-dimensional (1D)—neck feathers of domestic pigeons; (**B**) 1D—wings of Morpho butterflies; (**C**) Two-dimensional (2D)—barbules of male peacocks; (**D**) 2D—iridescent setae from Polychaete worms; (**E**) Three-dimensional (3D)—green spots in the wings of Parides sesostris butterfly; (**F**) 3D—Lamprocyphus augustus beetle. Adapted from [26] under CC BY 4.0 License.

Nature offers many examples of photonic crystal structures that have attracted a lot of attention due to their vivid structural colors caused by Bragg diffraction. Examples range from crystals, like opals, to living organisms, such as those shown in Figure 2: Neck feathers of domestic pigeons and wings of Morpho butterflies (1D PhC); barbules of male peacocks and iridescent setae from Polychaete worms (2D PhC); green spots in the wings of Parides sesostris butterfly and Lamprocyphus augustus beetle (3D PhC) [26].

As evident in these cases, the color is obtained thanks to microstructural periodic features; in fact, the presence of the periodicity results in constructive interference of the light reflected by the structures, depending on the geometry and the optical properties of the materials. The observation that in some cases these structural colors may change has led to the conclusion that an appropriate choice of responsive materials, which display structural modifications as a function of an applied stimulus, may pave the way to the development of several types of sensors.

According to the general definition that a mechanochromic effect occurs when a material or a structure changes its color in response to mechanical forces (typically; compression, stretching, shearing), these sensors may be labeled as mechanochromic PhC sensors. As a matter of fact, photonic crystals coupled with responsive materials and proper functionalization are able not only to select, but also to visualize, by means of the variation of the structural color, several parameters and species such as temperature, vapors, mechanical stress, chemical reagents and biomolecules. 

**Figure 3:**
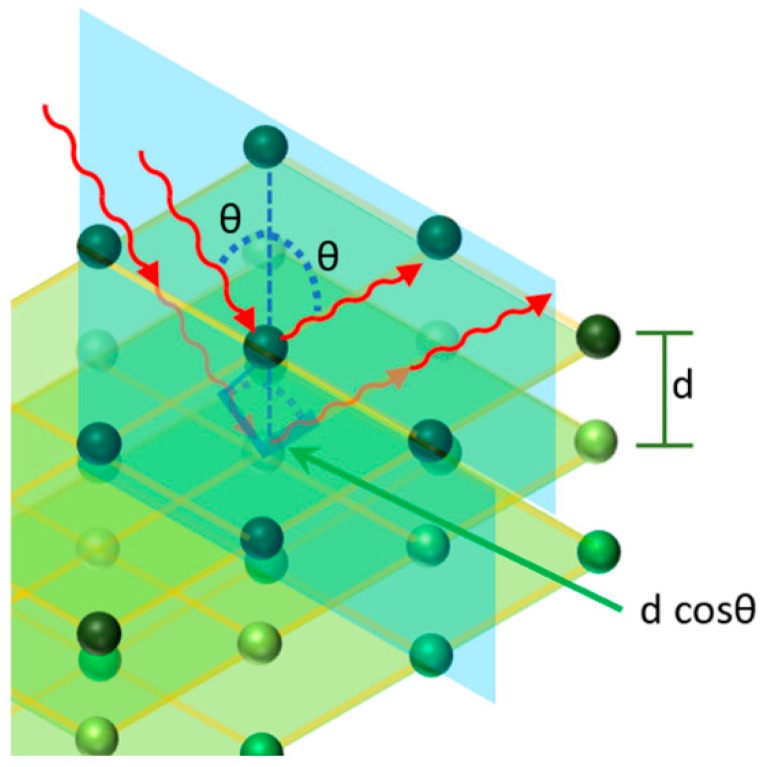
Scheme of the light reflection from ordered spherical particles by analogy with X-ray diffraction, where different wavelengths are diffracted at different angles.

Let us now briefly summarize the operation principle of these simple PhC structures. Colloidal crystals can be considered analogue to atomic crystals, and their structural colors can find explanation in the diffraction phenomenon (see Figure 3).

According to Bragg’s law of diffraction, which offers the rules of constructive interference and is expressed by Equation (1), where *d* is the distance between the atomic planes, *θ* and λ are the angle and wavelength of incident light, and *m* is the order of diffraction, one has:

(1) m λ=2 dcosθ

Let us consider a particular photonic crystal, namely a colloidal crystal array, which is constituted by dielectric spheres embedded in a dielectric medium, such as air or a different one. The combination of Bragg’s law and Snell’s law of refraction gives Equation (2), where *d* represents the distance between particle planes, *n_eff_* is the mean effective refractive index (RI), *θ* and *λ* are angle and wavelength of the reflected light, respectively, and m is the order of reflection:

(2) $$m\;\lambda =2\;d\;{\left(n_{eff}^{2}-\sin^{2}\theta \right)}^{1/2}$$

Moreover, it is possible to calculate the reflected wavelength considering the center to-center distance *D* between the spheres. The application of this method leads to Equation (3):

(3) $$m\;\lambda =2\cdot \sqrt{\frac{8}{3}}\cdot D\cdot {\left(n_{eff}^{2}-\sin^{2}\theta \right)}^{\frac{1}{2}}$$

Equation (4), where *n_p_* and *n_m_* are the refractive indices of the spheres and the surrounding medium, respectively, and *V_p_* and *V_m_* are the respective volume fractions, defines the mean effective refractive index *n_eff_*:

(4) $$n_{eff}^{2}=n_{p}^{2}\cdot V_{p}+n_{m}^{2}\cdot V_{m}$$

**Figure 4:**
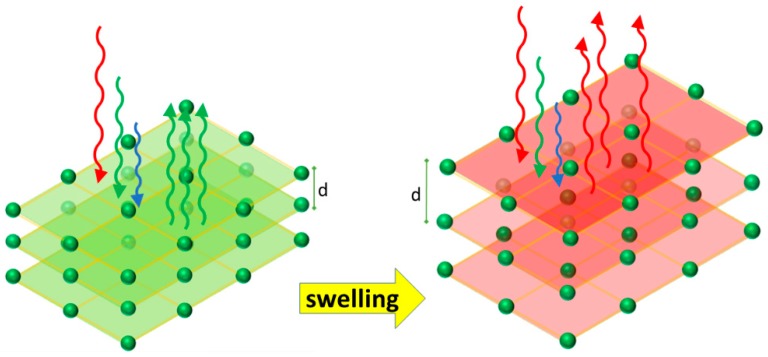
Change of the optical chromatic response, moving from green to red, of a 3D photonic crystals sensor due to an increase of the interplanar distance.

Focusing the attention on sensing phenomena, interesting kinds of sensors can be developed when the reflected or transmitted wavelength can be affected by external stimuli. In fact, focusing the attention on Equation (2), it is clear that a variation in the distance of the planes and/or in the effective refractive index induces a modification of the wavelength of the reflected light, as represented in Figure 4. Thus, the two main working mechanisms underlying the development of PhC optical sensors are either a variation of the refractive index of the system due to an external stimulus, such as absorption or immobilization of chemical and biological species, or a structural modification involving a change in the planes interdistance *d* (see Figure 4), e.g., due to absorption of chemical species (swelling) or to a stimulus such as mechanical stress. In practice, it may occur that both particles’ distance and refractive index vary simultaneously as a consequence of stimulus exposure; it has been shown, however, that the relative change in the distance d has more effect than the change in the refractive index on the shift of the wavelength of reflected light [27].

It may be underlined that, in many cases, the sensing PhCs do not exploit any photonic bandgap phenomenon; as explained above, the label of photonic crystals is due to their regular periodical structure, and their operation may be explained by classical Bragg diffraction (see equations above).

To give a broader overview, we must add that, in the literature, there are several examples of photonic crystals sensors based on fibers and waveguides as signal transducers. In this case, the signal is correlated to the change in the refractive index of the medium surrounding the guided-wave structure, e.g., a chemical component; using a proper functionalization of the fiber surface, even biomolecules, like proteins and nucleic acids, can bond to the surface and therefore induce a change of refractive index [24,25,28,29]. An enhancement of the detection sensitivity may be achieved by more complex systems, where one exploits the properties of a PhC structure and the surface plasmon resonance (SPR) phenomenon [30], or even a combination of magneto-optic and SPR effects [31]. An alternative to SPR and surface plasmon polaritons (SPP) is to exploit the excitation of Bloch surface waves (BSW) at the surface of a dielectric 1D photonic crystal—a sensor of this type was used for the label-free monitoring of human IgG/anti-IgG recognition [32]. Of course, all these structures are extremely efficient for the detection of tiny amounts of analytes (low limit of detection—LOD), but, in comparison with the colorimetric ones, they imply high costs for both their fabrication and the read-out of the signal. 

On the contrary, when employing chromatic structures, the detection is based on a visual response of the sensor, potentially avoiding any signal transduction; hence, this characteristic could favor the diffusion of these systems as simple and safe devices usable by untrained end-users in different applications fields.

It can be easily understood that, to boost the development of colorimetric sensors for different technological applications, it is necessary to create responsive artificial materials characterized by good selectivity, fast response rate, and excellent sensitivity. We may add that, as a relatively recent trend in the materials science field, the design and fabrication of PhCs with peculiar structural colors has also borrowed from nature (e.g., from the examples shown in Figure 2) [26,33,34].

## 3. Photonic Crystals for Chemical Sensing 

The simplest photonic crystal is represented by a 1D structure consisting of Bragg stacks based on multilayers film. With a proper choice of the constituting materials, even this basic structure has been successfully applied, similarly to more complex 2D and 3D PhCs, to a wide range of chemical and physical sensors. 

Let us present some examples of PhC sensors according to their application area and let us begin with chemical sensors, progressing from 1D to 3D. For a general review of PhCs for chemical sensing and biosensing, the reader is referred to [1]. A sensor based on the analysis of the wavelength shift of the Bragg‘s peak respect to the initial position was reported by Ghazzal et al., who used a multilayer film, constituted by mesoporous layers of silica and titania, for the discrimination of different solutions containing hydrophobic molecules (n-hexan) or hydrophilic ones (water) [35]. Colusso et al. [36] fabricated a structurally-colored hybrid silk-titanate 1D Bragg mirror, inspired by the cuticle of a beetle (Hoplia coerulea), and exploited the swelling properties of the silk component to measure the relative humidity (RH), with a dynamic range of 50 nm in the range between 10 and 80 RH%. The color change was reversible, and the structure showed good performances in terms of reproducibility and stability over time.

Focusing the attention on all-polymer Bragg multilayers, Lova et al. [37] developed a confined structure able to selectively detect organic volatile compounds (VOCs). With respect to the previous approaches, in this case the optical detection takes advantage of the effective diffusion of the analytes within the multilayered polymer film, and dynamic optical measurements may be performed. In fact, exploiting the characteristic chemico-physical interaction between the VOC and the polymer it is possible to obtain a specific and unique response associated to the kinetic process of the analyzed vapors. Specifically, the different spectral shift, acquired as a function of time, constitutes the fingerprint response peculiar of the investigated chemical. Thus, using a 1D PhC structure and exploiting dynamic optical measurements, that group developed a Flory-Huggins photonic crystal sensor for the on-site detection of pollutants [38]. 

**Figure 5:**
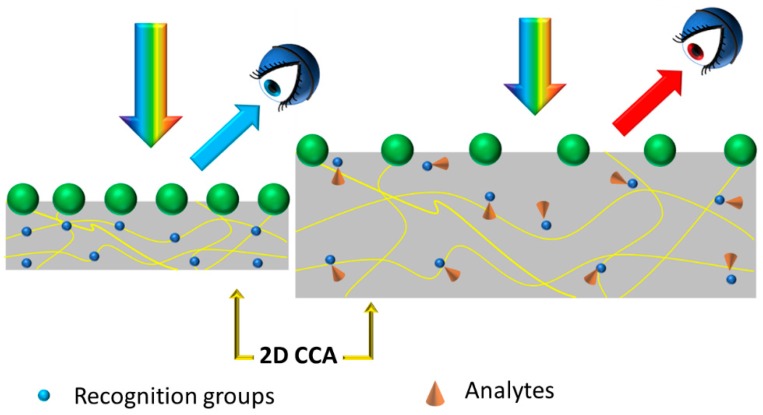
Working principle of the 2D colloidal crystals arrays (CCA) as responsive chromatic sensor: In this specific case, the interaction between analytes and recognition groups produces a swelling in the responsive material, inducing a modification of the particles spacing, and therefore a variation in the diffracted wavelength. Adapted with permission from [39].

Moving to 2D PChs, colloidal crystals are one of the most popular configurations among the 2D photonic structures. Two-dimensional hexagonal array monolayers can be created by exploiting self-assembly methods that allow organizing nano and microparticles in ordered structures. By combining such a structure with a responsive material, namely a material that has one or more properties that can be easily and significantly changed by an external stimulus (e.g., stress, temperature, humidity, pH, etc.), it is possible to design an efficient sensor. As an example, a stimulus changing the optical (e.g., the refractive index) or the structural (e.g., the thickness) properties of the responsive material induces a change in the features of the photonic crystal and therefore in its optical response (e.g., the wavelength of the reflected light), hence allowing the production of a low-cost chromatic sensor. Generally speaking, in the case of 2D responsive photonic crystals, when constituted by colloidal crystals arrays, they can be functionalized with a molecular recognition agent, which interacts with the analyte, thus producing a swelling that modifies the colloidal crystal spacing and in turn induces a shift, as evidenced in Figure 5, in the diffracted wavelength and a variation of the diffracted color.

Thus, the diffraction from 2D arrays can be used to optically monitor the responsive material volume variation. A review of 2D PhC sensors based on responsive polymer hydrogels chemically functionalized and used for the detection of many chemical and biomolecular analytes was presented in reference [39]. A possible approach to optically detect the response induced by the external stimuli makes use of the so-called Littrow configuration, where the incidence and diffraction angles are made to coincide, and the detection occurs at a specific angle by means of a spectrophotometer with a reflection probe [39]. A problem, however, is related to the fact that in a 2D PhC, as sketched in Figure 5, the number of diffractive elements is finite, which leads to a broadening of the reflected peak, causing a reduced sensitivity.

**Figure 6:**
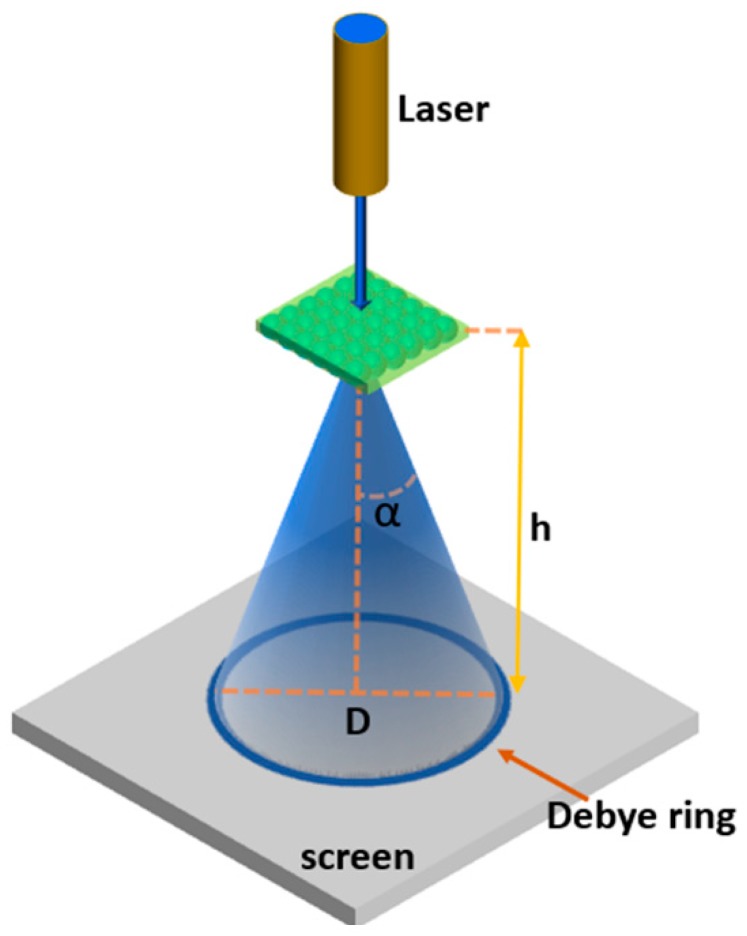
Schematic illustration of the Debye diffraction ring pattern produced by a 2D photonic crystal (PhC).

A possible solution to this issue concerns the measurement of the Debye diffraction ring diameter; this method avoids the use of sophisticated and expensive equipment and is suitable for not ‘highly’ trained personnel. One can look at the ring on a screen which is formed by the forward diffracted beam for normally incident monochromatic light, as sketched in Figure 6; the diffracted rings are dispersed radially. The Debye diffraction of a 2D array follows the law:

(5) $$\sin\alpha =\frac{2\cdot \lambda }{\sqrt{3}\cdot d}$$

where α is the forward diffraction angle of the Debye diffraction, *λ* is the incident wavelength, and *d* is the adjacent particle spacing. The forward diffraction angle, α, can be obtained from:

(6) $$\alpha =tan^{-1}\left(D/2h\right)$$

where *h* is the distance between the 2D array and the screen, and *D* is the Debye diffraction ring diameter.

Therefore, the particle spacing of a 2D array or the pore spacing of a 2D colloidal crystals can be easily determined by measuring the Debye ring diameter *D* using Equation (7):

(7) $$d=\frac{4\cdot \lambda_{laser}\sqrt{{\left(D/2\right)}^{2}+h^{2}}}{\sqrt{3}D}$$

Applying the Debye ring approach, several types of sensors were developed, such as the pH-sensitive system demonstrated by Xue et al. [40], that presents a large dynamic range, from 620 to 668 nm, in a pH interval between 3.22 and 7.91. Another hydrogel pH sensor was implemented by Cai et al. [39] by crosslinking chitosan with glutaraldehyde; the diffraction maximum shifted from 535 nm at pH 7 to 645 nm at pH 5. Jia et al. [41] developed a full-color photonic hydrogel for pH and ionic strength sensing that presented a good reproducibility for batch preparation. More recently, Li et al. [42] created a 2D colloidal crystals array able to monitor urea and urease inhibitor phenyl phosphorodiamidate (PPD), with a detection limit for urea and PPD of 1 mM and 5.8 nM, respectively; detection was made by measuring the diameter of Debye diffraction ring. The clear advantage of this approach is that one can even avoid using a spectrometer; a laser diode, a precise ruler, and the naked eye or a simple camera are sufficient.

Qi et al. [43] developed a portable device for the detection of Dipterex, an organophosphate pesticide, based on a 2D PhC fabricated starting from 600 nm polystyrene colloidal particles embedded into a polyacrylamide-acrylic acid hydrogel, and achieved a LOD of 7.7 × 10^−12^ mmol/L. As another example, Lan et al. [44], still using polystyrene particles but embedding them into a 3-acrylamidophenylboronic acid functionalized hydrogel, were able to realize a PhC sensor for glucose monitoring in urine. 

**Figure 7:**
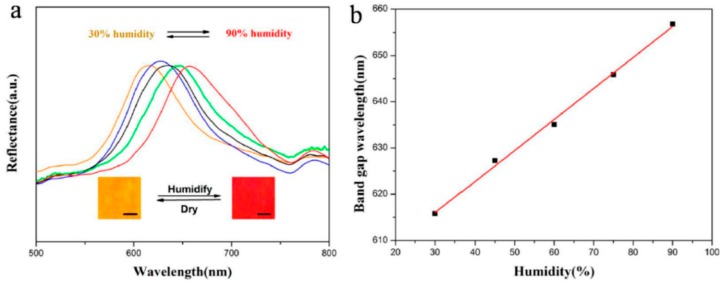
Response of a poly-ethylene glycol (PEG) hydrogel inverse opal as a humidity sensor: (**a**) UV-Vis reflection spectra at different RH% values (Insets show the color of the inverse opal sensor relative to 30% and 90% humidity; scale bars: 200 μm); and (**b**) band gap wavelength as a function of the humidity. Reproduced from [48] under CC BY 4.0 License.

Let us now discuss 3D colloidal crystal structures; their main advantage, which boosted their application in the sensors field, is that they allow the realization of low-cost, flexible, lightweight and power free systems for the analysis of different species in both liquid and gaseous forms. Besides opals, which also exist in nature, a variety of colloidal PhCs has been developed. The large surface area of inverse opals, for instance, has been widely exploited for the realization of gas sensors, and several examples can be found in the recent literature. Xing et al. [45] reached a detection limit of 200 ppb of acetone, by using a 3D inverse opals (3DIO) structure made of WO_3_ films prepared from a poly-methyl methacrylate (PMMA) latex sphere opal as a template, whereas Zhang et al. [46] reported a sensitivity of 100 ppb when using a 3DIO ZnO-Fe_3_O_4_ system. Both these sensors appear of potential use for noninvasive medical diagnosis, since acetone in human exhaled breath may be traced for diabetes monitoring. An In_2_O_3_ inverse opal was applied by Lee et al. to detect benzene, p-xylene, and toluene as indoor environmental pollutants [47]. In all the previously mentioned examples, the detection was based on electrical measurements, but the determination of some chemical and physical parameters can also be done optically. Yu et al., for instance, demonstrated the optical detection of relative humidity (RH%) using a poly-ethylene glycol (PEG) inverse opal chromatic sensor, the response being shown in Figure 7 [48]. If compared with traditional electrical humidity sensor, it has the great advantage of being not only economic but also a reversible naked-eye sensor.

**Figure 8:**
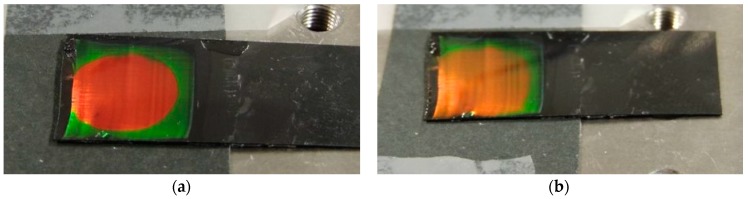
Chromatic response of a composite opal, originally appearing green, after spotting on the top surface 2 L of silicone fluid with different kinetic viscosity: (**a**) 10 cSt; (**b**) 1 cSt.

When considering the chemical sensing, both direct infiltrated opals and inverse ones have been used for the development of chromatic sensors. As an example of the first configuration, we fabricated a composite structure constituted by polystyrene nanoparticles and poly-dimethylsiloxane (PDMS), sensitive to different organic solvents (see Figure 8). Its working principle is based on the fact that the elastomeric matrix swells in a different way as a function of the solvent dropped on the surface of the sensor; this structure may also be used for the realization of a ‘reversible writing substrate’ [49].

**Figure 9:**
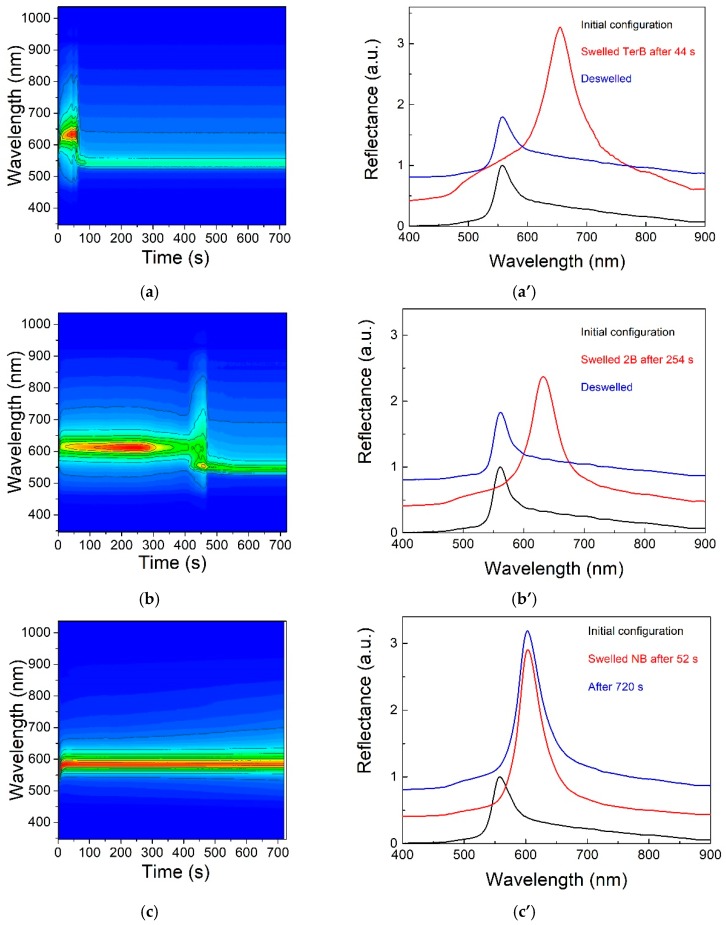
On the left, dynamic reflectance contour-plots spectra of: (**a**) 2-methylpropan-2-ol (TerB), (**b**) butan-2-ol (2B) and (**c**) butan-1-ol (NB), and, on the right, corresponding time-resolved optical responses (**a’**–**c’**). The measurements clearly show the differences typical of each isomer and the reversibility of the process. Reproduced with permission from [27].

Furthermore, with the aid of dynamic reflectance measurements, it was even possible to apply this structure to investigate mixtures, such as butanol-water, and to discriminate between homologues and isomers of butanol, characterized by similar physico-chemical features, thanks to the peculiar diffusivity and swelling properties of the matrix [29]. Figure 9, on the left column, shows the reflectance-time plots for three butanol isomers (TerB—2-methylpropan-2-ol, NB— butan-1-ol, and 2B— butan-2-ol, respectively); the vertical axis reports the wavelength, with the colors giving the normalized measured intensity, and the horizontal axis reports the time. Some reflectance spectra extracted from the previous plots are shown in the right column of the figure.

Inverse-opal sensors have been implemented using various materials; Kuo et al. achieved a sensitivity as high as 9100 nm/RIU in the detection of ethanol solutions, by employing titanium oxide [50]. A very interesting approach was used by Huang et al., who developed a label-free chromatic sensor for pesticide detection by using an inverse opal structure made of cross-linked poly(methacrylic acid) (PMAA) hydrogel and combining it with the molecularly imprinted polymer (MIP) technology. By imprinting methanephosphonic acid (MPA) sites in the PhC structure, the hydrogel particles showed a high specificity to MPA, achieving excellent limits of detection, down to 10^−6^ M. Moreover, the reflection color shift could be easily seen by naked eye [51]. 

It is evident that the inverse opal PhC structure, due to its high void fraction and consequent larger change of effective refractive index, is particularly suitable to the detection of gas and liquid substances and has therefore been adopted for many chemical and biochemical sensors [52,53,54,55,56,57,58,59,60].

As a help to the reader, the PhC chemical sensors presented in this section are summarized in Table 1, with the indication of the dimensionality (1D, 2D, or 3D), the structure’s material and the external stimulus or analyte detected. Unfortunately, the authors of the various papers do not report the performance of the respective sensor(s) by using a same parameter, e.g., the limit of detection (LOD). For this reason, the “Sensor’s response” column includes miscellaneous data, which are, in each case, representative of the corresponding sensor’s operation. As an example, the response of the sensor is often expressed by the change in resonance (diffraction) wavelength Δλ_B_ with respect to the Bragg peak’s wavelength; when direct visual detection is considered, a higher value of Δλ_B_ corresponds to a larger difference of the hue stimulus for the observer, hence better performance of the sensor. To permit some comparison between the given values of Δλ_B_ and the capability of chromatic detection by an observer, Table 2 reports the wavelength ranges assigned to various hue sensations by Tilton [61]. As expected, a same Δλ may give different hue sensations depending on the operational wavelength *λ* it is therefore important, in the experimental tests, to choose λ in such a way to enhance the change of hue sensation for small changes of λ.

It must be underlined that Table 1 and the following tables are intended to provide examples of application of different PhC structures to the sensing of various parameters, with the aim of being useful to newcomers in the field. These two tables cannot neither give any indication about the “best” sensor nor allow an easy comparison with traditional electronic or optical sensors. A comparison, in fact, is only possible when different sensors are used in the same environmental conditions (e.g., temperature, humidity) and the same instrumentation (e.g., light source and detector). As an example of the variability of results even for a same type of sensor, let us refer to acetone gas sensors, which are of particular interest for diabetes monitoring through the analysis of the human exhaled breath. Table 3 presents the results achieved with the two 3D PhCs listed in Table 1 and with other metal-oxide based sensors [62]. It appears clearly that the LOD is quite different among different sensors, and that the performance of the two PhC sensors is better than most of the other sensors. It must be kept in mind, however, that the choice of a sensor is also depending on many other factors, often related to the specific application: Simplicity, cost, robustness, disposability, etc.

It may be noted that the two 3DIO sensors in the Table 3 are capable of working at relatively low concentrations of acetone (5 and 50 ppm, respectively), and are therefore superior to most commercially available sensors, that work in the concentration range 50 to 500 ppm. Therefore, only few sensors, including the WO_3_ 3DIO, are suitable for human glucose monitoring application, since the exhaled acetone approximately ranges from 0.2 to 25 ppm in healthy people and people with diabetes, respectively [62].

## 4. Photonic Crystals for Biological Sensing

Similar to what was reported in the previous section, photonic crystals with different geometrical configurations are a suitable tool for the development of biochemical and biological sensors, too. 

As an example of a 1D PhC sensor, Bonifacio et al. [79], exploiting a Bragg structure made of functionalized mesoporous multilayers of alternating refractive index, realized a platform to be used as photonic nose for the identification of volatile chemicals and biological elements. This PhC architecture is characterized by a large surface area and, upon infiltration and capillary condensation of solvent vapors, produces color changes that may be easily monitored by reflectivity or transmissivity measurements. As a proof of the potential utility in the area of disease diagnostics, detection of bacteria Pseudomonas aeruginosa, Escherichia coli, Staphylococcus aureus, and Staphylococcus epidermidis was successfully performed.

Referring to 2D PhC sensors, one can mention the results by Qi et al [80], who developed a tunable acetylcholinesterase (AChE)-functionalized 2D structure for the detection of a real nerve agent, sarin, and observed a linear relationship between the logarithm of the sarin concentration and the particle spacing of the 2D PhC in the range from 7.1 × 10^−14^ to 7.1 × 10^−1^ mmol/L. The same structure could be the basis for sensing of other G-series nerve agents. 

Looking at the recent literature, one can find several examples of 3D colloidal crystals used for the development of biosensors; in particular, hydrogels have been widely employed for biomolecules detection. Feng et al., for instance, synthetized an innovative hydrogel formed by a mixture of 3-aminophenylboronic acid (APBA), cross-linker ethyleneglycol dimethacrylate (EGDMA), radical initiator azobisisobutirronitrile (AIBN) and methanol for the fabrication of a highly selective colorimetric glucose sensor, able to discriminate among glucose and other carbohydrates with 1,2-cis-diol groups [81]. A polyethylene glycol diacrylate (PEG-DA) nanoporous hydrogel colloidal crystal, reported by Choi et al. [82], allowed the determination of a concentration of immunoglobulin G antibody of 10 mg/mL by simply observing a chromatic variation in the opalescence. 

**Figure 10:**
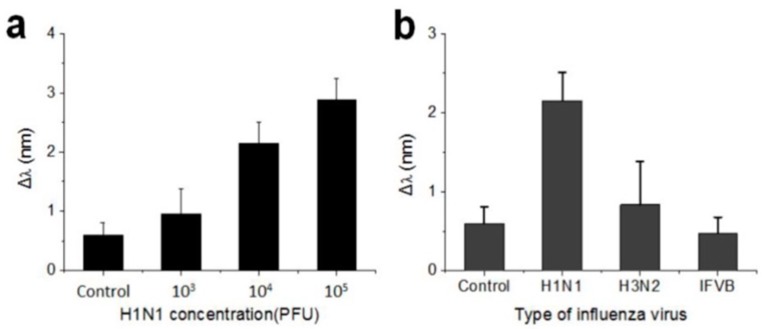
(**a**) Shift of reflectance peak as a function of H_1_N_1_ virus subtype concentration, for concentrations ranging from 10^3^ to 10^5^ PFU in 10 L. Phosphate-buffered saline (PBS) solution is used for the control; (**b**) Shift of the reflectance peak depending on the type of virus, for a concentration equal to 10^4^ PFU for all influenza viruses, namely A (H_1_N_1_), A (H_3_N_2_), and B (IFVB). Specificity to the H_1_N_1_ is evident. Reproduced from [83] under CC BY 4.0 License.

Inverse opal structures have been widely used for the implementation of PhC biological sensors, too. As an example, an antibody-immobilized silica-based inverse opal nanostructure was successfully used to label-free detect influenza viruses, with a selectivity for H_1_N_1_ subtype and a sensitivity in the range of 10^3^–10^5^ plaque forming unit (PFU) [83]. Both the selectivity and the sensitivity have been determined through the measurements of the red shift of the reflectance peak (see Figure 10). The authors, thus, proposed a generalized simple-readout sensing platform for biohazards, where the surface functionalization of the nanostructure may be exploited for different sensing strategies.

Inverse opal hydrogel particles, which also resulted ideal enzymatic carriers for biocatalysis, were synthetized by Wang et al. [84], who exploited their structural colors and variation in the Bragg peak’s wavelength induced by the increased average refractive index of particles after enzyme attachment.

Inverse opals have also found application in the development of fluorescence-based biosensors: Chiappini et al. [85] reported a study and some preliminary results on tumor necrosis factor (TNF) recognition, employing a silica inverse opal taking advantage of the DNA-aptamer-Cy3 immobilization. Lee et al. used a specific functionalization for streptavidin by micropatterning biotin on the inverse opal hydrogel to reach higher fluorescence and limit of detection of 1.0 nM [86]. A review of analyte-sensitive photonic crystal hydrogel sensors, indicating potential applications in clinical sampling, was published by Yetisen et al. [87].

As done for chemical sensors, here, a summary of the PhC structures for biological sensing presented in Section 4 is shown (see Table 4). Again, the “Sensor’s response” column provides heterogeneous data, as reported in the articles cited, which, in each case, are representative of each sensor’s operation. 

It may be worth underlining that the peculiar structural features of 3D colloidal crystals make them interesting not only for sensors but also for complementary applications in the biological field, such as cell scaffold and drug delivery. In the recent literature, for instance, one can find a review paper by Zhang et al. [88] reporting the employment of inverse opal scaffold for regenerative medicine, pointing up the crucial role of this kind of uniform porous structures. Zhu et al. [89], too, highlighted the importance of the structural features in terms of uniform domains and regular interconnectivity of the pores for the use as cell scaffolds. Xiao et al., to study the topographical influence on mesenchymal stem cells (MSCs) and the cell-substrate interaction, used an elastomeric inverse opal structure to obtain a well-defined cell orientation [90]. Even the structural voids of 3D photonic structures play a fundamental role not only in sensing applications: in fact, they allow loading and subsequent releasing, under controlled conditions, of specific molecules, which is crucial for the use of 3D colloidal crystals as drug delivery systems. The release of the chemical species may be controlled or adjusted by the variation of some parameters such as the pH [91] or the temperature [92], or by electrical stimulation [93].

## 5. Mechanochromic Sensors Based on Photonic Crystals

When considering the field of mechanochromic sensors, i.e., structures changing their color in response to mechanical forces (typically; compression, stretching, shearing), it is worth mentioning that great effort has been produced in the last years to develop mechanical sensors characterized by low production costs, low power consumption and, last but not least, easiness of interrogation. Although several materials and approaches have been employed, the most used ones are metallic nanoparticles or pigments. Concerning the different working principles, they are based on bioluminescence or periodic spatial structures (photonic crystals). In the first group of sensors, the colors are caused by specific absorption of light by metallic nanoparticles (NPs) or molecules embedded in the matrix, as demonstrated by Jiang et al. [94] and Duarte et al. [95], respectively. When based on bioluminescence phenomenon, the sensing signal is produced as a consequence of chemical reactions in the photophores of some organism [96]. Otherwise, the color can be caused by interaction of the incident light with periodic structures—as described in Section 2, the color displayed by periodic structures can be described according to the Bragg’s law (see Equation (1)).

Looking at recent literature, according to Chan et al. [97], the main systems employed as PhC mechanochromic sensors are 1D and 3D structures, which are easier to fabricate than the 2D ones, that would require nanoimprinting or colloidal crystals lithography [98,99].

As for 1D systems, one of the simplest ones is based on submicron wrinkling shape grating. Yu et al. [100] managed to fabricate a flexible tunable optical grating: Following the modification of the periodic sinusoidal pattern as a consequence of mechanical deformation, they noticed a shift of 85 nm of the first order diffraction peak when stretching the structure of 30%. In this case, it is important to highlight that the periodicity of the buckling pattern can be described by Equation (8), as shown in [101]:

(8) $$\lambda =2\pi t\cdot {\left[\frac{\left(1-\nu_{s}^{2}\right)E_{f}}{3\left(1-\nu_{f}^{2}\right)E_{s}}\right]}^{1/2}$$

where *λ* is the wavelength of the grating buckling-periodic-pattern, *E_f_* and *ν_f_* are the elastic modulus and the Poisson’s ratio of the film, *E_s_* and *ν_s_* are the elastic modulus and Poisson’s ratio of the substrate, and *t* is the thickness of the film. An extended discussion of the buckling instabilities in periodic composite materials, which are especially important for flexible electronics and photonics, is reported in [101].

**Figure 11:**
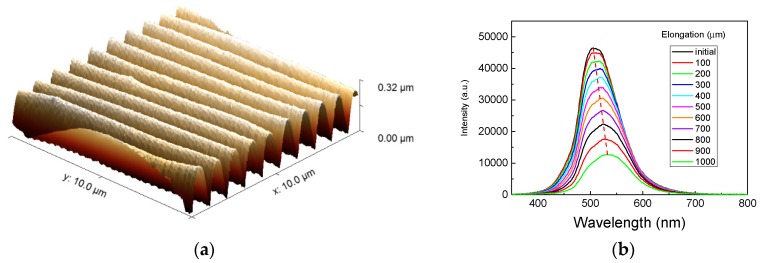
(**a**) Atomic Force Microscope (AFM) image showing large ordered regular arrays, with typical dimension of 50 µm × 50 µm, with a periodic pitch of about 1µm and amplitude of the wrinkle of ~ 200 nm. (**b**) Intensity of the transmittance diffraction at different applied strain levels.

More recently, following the same approach, Piccolo et al. [102] produced a mechanochromic strain sensor based on 1D wrinkled structures (see Figure 11), where the application of a longitudinal strain induced a variation in the optical response with a red shift of the position of the diffraction peak and a decrease in its intensity due to the change in amplitude of ripple of the grating with applied strain. In fact, the performance of the device in terms of sensitivity was around 10 nm/%, which is comparable with those reported in the literature [103].

Following a different approach, Minati et al. developed a metallic-dielectric chromatic structure by combining self-assembly and peeling off techniques, obtaining 300 nm periodic stripes of self-assembled gold nanoparticles [103]. The aligned Au nanoparticles array was characterized by high reflectance in the visible range and optical properties similar to that of an optical grating. In terms of sensitivity, the system exhibited a value of 5.2 ± 0.1 nm/% when stretched up to 18% of its initial length. 

A completely different kind of mechanochromic 1D photonic crystal, constituted by an anisotropic hydrogel, was presented by Haque et al. [104]. Their photonic crystal showed a high mechanical strength and very good fatigue resistance; it was fabricated by embedding hydrophobic poly(dodecyl glyceryl itaconate) bilayers in a polyacrylamide matrix.

Moving to 2D photonic crystals, chromatic sensors have been produced taking advantages of two complementary approaches: Nanoimprinting and colloidal crystal lithography. In the first case, Endo et al. [105] showed that is possible to fabricate two-dimensional PhCs on a cyclo-olefin sheet using a printable photonics technology. More recently, the same group [106], still using two-dimensional PhCs, was able to develop a sensor for the detection of C-reactive protein CRP with low non-specific adsorption using an antigen-antibody reaction characterized by an extremely short reaction time.

A low-cost technology based on oxygen plasma treatments to fabricate orthogonal diffraction gratings on the two sides of a poly-dimethylsiloxane film was reported by Guo et al. [107] for the determination of vectorial strain/stress exploiting Fraunhofer diffraction. The interesting characteristic of this sensor is its capability to display and assess the stress/strain in both x and y directions, through the analysis of both position and intensity of diffraction spots. 

**Figure 12:**
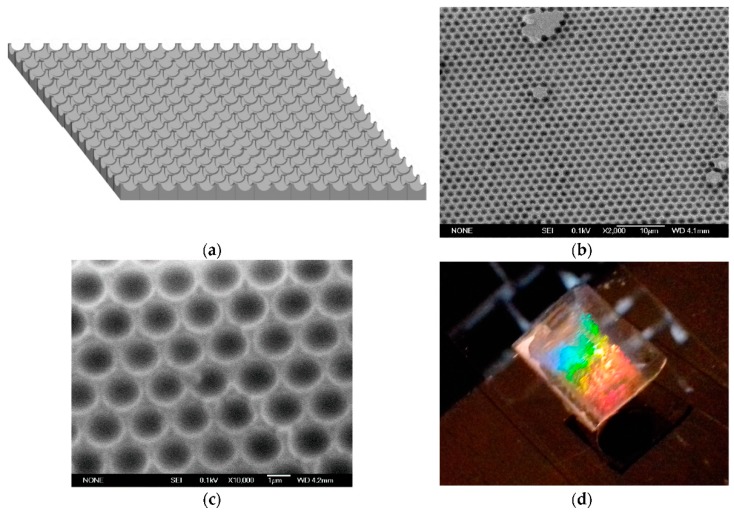
(**a**) Sketch of the concave structure obtained via soft lithography (not in scale). (**b**) Scanning electron microscopy (SEM) surface image of PDMS inverse colloidal crystal. (**c**) SEM detail of the ordered hexagonal array. The bar corresponds to 1 µm. (**d**) Diffracted light from the hexagonal array of voids on transparent elastomeric substrate. Reproduced from [108] under CC BY 4.0 License.

Another example of chromatic 2D system, obtained by colloidal crystal lithography, was proposed recently by Piccolo et al. [108,109], who developed a strain/stress vectorial sensor based on quasi-hemispherical voids in a hexagonal arrangement on a transparent flexible PDMS substrate, as shown in Figure 12. In this case, too, it is possible to acquire the vectorial strain-stress information in the two directions by means of the analysis of the displacement of two different diffraction spots.

In a very recent paper, Zhao et al. [110] reported an elastic, stretchable, photonic crystal characterized by periodic cylinder-shaped air holes in triangular lattice. The mechanochromic response was enhanced by the particular geometric configuration, capable of undergoing up to 2000 stretching cycles of deformation without degradation, and with a color change ranging all the visible wavelengths with a low 29% stretching rate.

**Figure 13:**
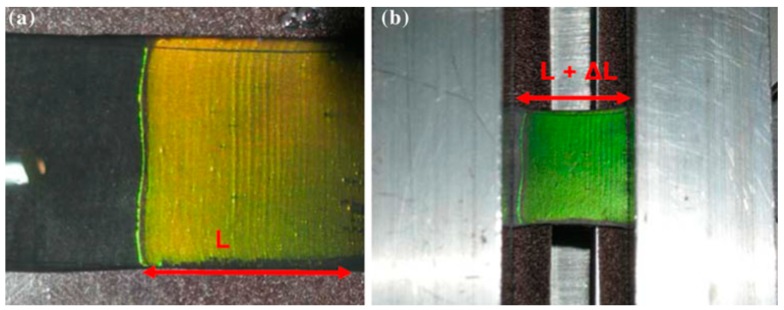
Observed changes in the structural color of the colloidal crystal film deposited on a Viton substrate (1.6 × 1.6 cm^2^). (**a**) Photo of the initial sheet (length L); (**b**) photo of the stretched sheet (L + DL; DL = 2 mm). Reproduced with permission from [113].

When speaking about 3D structures, a few examples can be cited addressing the topic of mechanochromic sensors. The pioneers in this field were Fudouzi et al. [111,112], who developed a polymeric sensor constituted by polystyrene spheres embedded in an elastomeric matrix; when the structure was subjected to a mechanical deformation, they detected a linear shift of the diffraction peak. Later, Chiappini et al. [113], by increasing the interplanar distance of the polymeric beads, managed to enlarge the dynamic range to a value greater than 30 nm. As one can easily see in Figure 13, their photonic crystals where engineered to display a visible color change under ≈ 10% of elongation. The fabricated structure presented a linear response and high reversibility under several deformation cycles; it was envisaged to apply this sensor to the detection of cracks on concrete bridges. 

More recently, Ge et al. [114] proposed an innovative fast responsive system, based on a mechanochromic gel constituted by SiO_2_ spheres embedded in a photopolymerized mixture of ethylene glycol and poly(ethylene glycol)methacrylate (PEGMA). Keeping in mind the application to record pressures in the MPa range, correlated to traumatic brain injuries, Yang et al. [115] managed to reach a mechanochromic sensitivity (MS) Δλ/Δε of 5.7 nm/% employing an un-crosslinked SU-8 inverse opal. In this regard, it is useful to recall that the MS represents the shift in wavelength as a function of the applied strain, and, as reported by Chan et al. [97], strongly depends on the materials employed, with values ranging from 0.7 to 5.7 nm/%. 

Another very recent work that is worth mentioning was performed by Snapp et al. [116], who successfully merged a 3D photonic crystal and a graphene monolayer stretchable transducer, fabricating a compact, highly sensitive, electrically measurable colorimetric sensor for the structural health monitoring field. The strong point of their work is the combination of the photonic crystal that allows a direct visual perception of strain and the 2D conducting material that enables the strain quantification via electrical measurement.

## 6. Conclusions

The unique properties of photonic crystals proved to be very important, among several other applications, for the development of advanced sensing devices. A search in the Clarivate Web of Science database, using photonic crystal* and sens* as topics, produced more than 16,000 results, and almost one half of them (7948) were published in the last five years. This great interest has been boosted by the growing commercial demand of sensors in many areas; according to a recent review [117] and a market analysis by Allied Market Research [118], the global photonics sensor market is expected to attain $ 18 billion by 2021, with a compound annual growth rate (CAGR) of 17.7% in the period 2016–2021; moreover, the global biophotonics market, which includes many other applications besides biosensors, is estimated to reach $63.1 billion by 2022 [119]. 

The goal of this review was to provide to both the newcomers in the field and the experts a general overview of 1D, 2D, and 3D PhC structures which have been proposed and demonstrated as effective sensors, so to attract their attention and possibly inspire new approaches. We hope having succeeded in it, even if here we have been able to mention only a small fraction of the published results. As an example, we have also shown how PhC sensors compare well with other types of sensors in the detection of acetone, which is of interest for glucose monitoring through the analysis of the human exhaled breath.

An emerging R&D line, which is rapidly growing in parallel to flexible electronics [120,121,122] and flexible photonics [123], concerns the development of flexible PhC sensors, especially of those based on the visual readout of the change of structural colors induced by an external stimulus. In this area, an interesting result was recently presented by Tsuchiya et al. [124], who used colloidal crystal hydrogel microbeads and took advantage of the fact that the ordered arrangement of microspheres or hemispherical domes exhibit structural colors with low angle dependency [125]. The authors claim that, as the stimuli-responsive microbeads are freely dispersed onto a flexible sheet, unexpected color changes with the mechanical deformation caused by the bending or stretching of the flexible sensing device are avoided. Whilst mechanochromic PhC sensors are attracting an ever-increasing interest, since they constitute a simple, low-cost, and effective solution especially for monitoring bulk materials and structural health, adding mechanical flexibility may further enhance and broaden their applications [98].

Overall, the prospect of the field of PhC sensors, especially of those based on chromatic response to external stimuli, is undoubtedly bright, and their appearance on the market should not be delayed too long.

## Figures and Tables

**Table 1:** Summary of the photonic crystal structures for chemical sensing presented in Section 3. The first column (left) indicates the dimensionality of the PhC, whereas the second column indicates the type of structure and its constituting materials. ∆λ_B_ is the value of the shift of the Bragg peak’s wavelength. When available, the value of the limit of detection (LOD) is reported.

PhC	PhC Structure and Material	External Stimulus or Analyte Detected	Sensor’s Response	Ref.
1D	Mesoporous layers of silica and titania	Hydrophobic and hydrophilic molecules	∆λ_B_ = 47 nm for DHDP in THF	[35]
1D	Hybrid silk-titanate layers	Humidity (RH)	∆λ_B_ = 40 nm sensitivity (∆λ /∆RH%) = 0.28	[36]
1D	All-polymer Bragg multilayers (PPO, CA)	Volatile organic compounds	Chromatic discrimination of benzene, ODCB, CT, toluene	[37]
1D	All-polymer Bragg multilayers (PS, CA)	Pollutants	LOD: MeOH (29 mg/L); EtOH (12 mg/L); 1 POH (12 mg/L); 2 POH (6 mg/L); BuOH (64 mg/L)	[38]
2D	Composite Hydrogel (PS, poly HEMA-AA)	pH	∆λ_B_ = 130 nm when pH goes from 2 to 8	[40]
2D	Composite hydrogel (Fe_3_O_4_ NPs embedded in PAM-PAA)	pH and ionic strength	∆λ_B_ = 202 nm when pH goes from 2.02 to 7.03; ∆λ_B_ = 181 nm when NaCl varies from 0.02 M to 1 M	[41]
2D	Composite Hydrogel (PS, AM)	Urea and urease inhibitor	∆λ_B_ = 28 nm when urea concentration varies from 1 to10 mM	[42]
2D	Composite Hydrogel (PS, PAM-AA)	Organophosphate pesticide	LOD: 7.7 × 10^−12^ mmol/L for Dipterex	[43]
3D	WO_3_ inverse opal	Acetone	LOD: 0.2 ppm	[45]
3D	ZnO-Fe_3_O_4_ inverse opal	Acetone	LOD: 0.1 ppm	[46]
3D	PEG inverse opal	Humidity	∆λ_B_ = 40 nm when RH% goes from 30 to 90	[48]
3D	Composite direct opal based on PS and PDMS	Organic solvents	Chromatic discrimination of EtOH, MeOH, Silicone fluids	[49]
3D	Composite direct opal based on PS and PDMS	Butanol isomers	Chromatic discrimination of TerB, NB, 2B	[27]
3D	Titanium oxide inverse opal	Ethanol solutions	Sensitivity = 9090 nm/RIU	[50]
3D	MIP-hydrogel particles	MPA	Detection down to 1×10^−6^ M	[51]

Abbreviations: DHDP: dihexadecyl phosphate; THF (solvent): tetra-hydrofuran; RH: relative humidity; PPO: poly(p-phenylene oxide); CA: cellulose acetate; ODCB: 1,2-dichlorobenzene; CT: carbon tetrachloride; PS: polystyrene; MeOH: methanol; EtOH: ethanol; 1 POH: 1-propanol; 2 POH: 2-propanol; BuOH: 1-butanol; HEMA: 2-hydroxethyl methacrylate; AA: acrylic acid; NP: nanoparticle; PAM: poly(acrylamide); PAA: poly(acrylic acid); AM: acrylamide; PEG: polyethylene glycol; PDMS: polydimethylsiloxane; TerB: 2-methylpropan-2-ol, NB: butan-1-ol; 2B: butan-2-ol; MIP: molecularly imprinted polymer; MPA: methanephosphonic acid.

**Table 2:** Hue sensation and corresponding wavelength range. Data extracted from [61].

Hue Sensation (Name of Color)	Wavelength Range (nm)	Range Width (nm)
Violet	388–429	41
Indigo	429–458	29
Blue	458–481	23
Cyan	481–499	18
Turquoise	499–513	14
Green	513–528	15
Lime	528–546	18
Chartreuse	546–561	15
Yellow	561–575	14
Lemon	575–587	12
Ocher	587–599	12
Orange	599–610	11
Tangerine	610–622	12
Ruby	622–636	14
Red	636–782	146

**Table 3:** Summary of the literature review of selected PhC and metal-oxide based sensors for acetone detection. The columns, from left to right, indicate the sensor’s material, the optimal operation temperature, the quantity of acetone (concentration) used for the test, the limit of detection (LOD), and the bibliographic reference. Adapted from [62].

Material	Temperature (°C)	Acetone (ppm)	LOD (ppm)	Reference
C_3_N_4_-SnO_2_	380	20	0.087	[63]
NiO/SnO_2_	300	50	0.01	[64]
WO_3_ 3DIO *	370	5	0.1	[45]
TiO_2_/In_2_O_3_	250	10	0.1	[65]
CuFe_2_O_4_/α-Fe_2_O_3_	275	70	0.1	[66]
ZnO–Fe_3_O_2_ 3DIO *	475	50	0.15	[46]
GO-SnO_2_-TiO_2_	200	5	0.25	[67]
Pt_0.3_Au_0.7_–In_2_O_3_	160	50	0.3	[68]
Co1*−*xZnx Fe_2_O_4_	650	50	0.3	[69]
WO_3_ NFs	350	5	0.4	[70]
Cr-doped CuO	450	3.2	0.4	[71]
SnO_2_/SiO_2_	270	300	0.5	[67]
ZnCo_2_O_4_	200	500	0.5	[72]
Ru/WO_3_	300	1.5	0.5	[73]
NiFe_2_O_4_	160	200	0.52	[74]
ZnO:Pt	400	1000	1	[75]
ZnO:Nb	400	1000	1	[76]
Pd/LaFeO_3_	200	1	1	[76]
WO_3_/Pt-GNs **	200	10	1	[77]
In/WO_3_-SnO_2_	200	50	1	[78]

* 3DIO: three-dimensional inverse opal: ** GNs: graphene nanosheets.

**Table 4:** Summary of photonic crystal biological sensors. The first column (left) indicates the dimensionality of the PhC, whereas the second column indicates the type of structure and its constituting materials. In the sensor’s response columns, Δλ_B_ is the value of the shift of the Bragg peak’s wavelength induced by the external stimulus or analyte (third column). When available, the value of the limit of detection (LOD) is shown.

PhC	PhC Structure and Material	External Stimulus or Analyte Detected	Sensor’s Response	Ref.
1D	Mesoporous multilayer Bragg stack (SiO_2_-TiO_2_ NPs)	Bacteria	Chromatic discrimination of ATCC 27853, ATCC 25922, ATCC 29213, ATCC 12228	[79]
2D	Composite hydrogel (PS, PAM-AA)	Glucose	Detection range from 0.4 to 53.3 mmol/L	[44]
2D	Composite hydrogel (PS, HEMA)	Sarin	LOD: 6.7 × 10^−14^ mmol/L	[80]
3D	hydrogel inverse opal (3-APBA)	Glucose	Δλ_B_ = 139 nm for glucose concentration of 5 mmol	[81]
3D	hydrogel inverse opal (PEG-DA)	Immunoglobulin G antibody	Δλ_B_ = 50 nm for a concentration of 10 mg/mL	[82]
3D	Inverse opal (functionalized Si)	Influenza viruses	Selectivity of H1N1 subtype; detection in the range 10^3^–10^5^ PFU	[83]
3D	Hollow hydrogel particles (AAm)	Enzyme	Chromatic detection of enzyme activity	[84]
3D	Inverse silica opal (functionalized)	Tumor necrosis factor (TNF)	Detection of TNF-alpha via fluorescence quenching	[85]
3D	Inverse opal hydrogel (PHEMA)	Streptavidin	LOD: 1.0 nM	[86]

Abbreviations: NPs: nanoparticles; ATCC27853: pseudomonas aeruginosa; ATCC 25922: Escherichia coli; ATCC29213: staphylococcus aureus; ATCC 12228: Staphylococcus epidermidis; PS: polystyrene; PAM-AA: polyacrylamide-co-acrylic acid; HEMA: 2-Hydroxyethyl methacrylate; 3-ABPA: 3-acrylamidophenyl boronic acid; AChE: acetylcholinesterase; PEG-DA: poly(ethylene glycol)-diacrylate; H_1_N_1_: influenza A subtype H_1_N_1_; AAm: acrylamide; TNF-alpha: tumor necrosis factor alpha; PHEMA: poly(2-hydroethylmethacrylate).

## Abbreviations

excluding chemical and biological substances

1D	One-dimensional
2D	Two-dimensional
3D	Three-dimensional
3DIO	Three-dimensional inverse opal
BSW	Bloch surface waves
CAGR	Compound annual growth rate
CCA	Colloidal crystal array
LOD	Limit of detection
MS	Mechanochromic sensitivity
NP	Nanoparticle
PBG	Photonic band gap
PCF	Photonic crystal fiber
PFU	Plaque forming units
PhC	Photonic crystal
RH	Relative humidity
RI	Refractive index
RIU	Refractive index unit
SEM	Scanning electron microscopy
SPP	Surface plasmon polaritons
SPR	Surface plasmon resonance
